# Meningeal-tumor interactions define distinct modes of leptomeningeal colonization in Group 3 medulloblastoma

**DOI:** 10.1186/s40478-026-02253-7

**Published:** 2026-04-01

**Authors:** Leyre Jimenez Garcia, Lindsay N. Ryan, Amy C. Gross, Annika M. Albrecht, Jefferson H. Walters, Jeffrey R. Leonard, Ryan D. Roberts, James B. Reinecke

**Affiliations:** 1 Molecular, Cellular, and Developmental Biology Program, The Ohio State University, Columbus, OH USA; 2 The Center for Childhood Cancer Research, Abigail Wexner Research Institute at Nationwide Children’s Hospital, Columbus, OH USA; 3 The Ohio State University, Columbus, OH USA; 4 Department of Neurosurgery, The Ohio State University College of Medicine, Columbus, OH USA; 5 Department of Neurosurgery, Nationwide Children’s Hospital, Columbus, OH USA; 6 Department of Pediatrics, The Ohio State University College of Medicine, Columbus, OH USA; 7 Division of Hematology, Oncology, and Bone Marrow Transplant, Nationwide Children’s Hospital, Columbus, OH USA; 8 The James Comprehensive Cancer Center, The Ohio State University, Columbus, OH USA

**Keywords:** Medulloblastoma, Leptomeningeal metastasis, Adhesion, Co-culture models

## Abstract

Medulloblastoma, the most common malignant brain tumor in children, shows a pronounced tendency to spread to the leptomeninges. Leptomeningeal metastasis accounts for nearly all medulloblastoma-related deaths, yet the cellular and molecular mechanisms driving this process remain poorly understood. Progress has been hindered by limited access to patient samples, the fragile anatomy of the leptomeninges, and the lack of robust preclinical models. Here, we developed an in vitro model that captures key features of the early leptomeningeal niche. We demonstrate that human meningeal cells promote medulloblastoma survival and proliferation under nutrient-deprived conditions both in vitro and in vivo. Using this system, we uncovered mechanisms that govern distinct patterns of leptomeningeal colonization in vivo. This physiologically informed and experimentally validated model offers a tractable platform for elucidating the molecular basis of leptomeningeal colonization and for identifying therapeutic vulnerabilities that can prevent medulloblastoma dissemination.

**Supplementary Information:**

The online version contains supplementary material available at 10.1186/s40478-026-02253-7.

## Introduction

Nearly all deaths from medulloblastoma, the most common malignant brain tumor of childhood, are due to leptomeningeal metastasis [1–5]. The biological mechanisms that enable tumor cells to survive and expand within the unique leptomeningeal compartment remain poorly defined [6]. Consequently, therapeutic strategies capable of eradicating disseminated tumor cells from the leptomeninges are lacking, particularly for patients who relapse after standard-of-care therapy [7].

The leptomeninges, comprising the pia mater, subarachnoid space (filled with cerebrospinal fluid—CSF), and arachnoid mater, were once viewed primarily as structural coverings to the brain and spinal cord but are now recognized as dynamic microenvironments that actively participate in central nervous system health and disease, including cancer [8–11]. Within the CSF, medulloblastoma cells exist in at least two states: free-floating within the fluid or adherent to the leptomeningeal surface of the brain and spinal cord. Adherent lesions may form diffuse, laminar coatings or discrete nodules along the leptomeninges, yet the mechanisms governing these distinct metastatic patterns remain unknown [12, 13]. This knowledge gap underscores the need to understand how interactions between tumor cells and leptomeningeal stromal elements influence colonization, survival, and progression.

Advances in leptomeningeal metastasis biology have been hindered by several experimental and clinical barriers [14]. Surgical access to leptomeningeal lesions is limited due to their diffuse nature and lack of therapeutic benefit from resection, precluding comprehensive molecular analysis of matched primary and metastatic samples. In the few available cases, metastatic lesions exhibit marked biological divergence from their primary tumors, underscoring the need to study the metastatic niche directly [15]. Moreover, while mouse models have been invaluable for identifying molecular drivers of medulloblastoma initiation, endpoints are reached due to the rapid growth of cerebellar tumors that cannot be surgically resected, not leptomeningeal metastasis [16]. Lastly, conventional in vitro models fail to replicate the cellular composition and nutrient-deprived microenvironment of the CSF, limiting their translational relevance.

Organotypic co-culture systems have proven valuable for modeling tumor–host interactions that occur during metastatic dissemination to lung and bone marrow in other solid tumor contexts [17–20]. Leptomeningeal stromal-tumor co-cultures have been applied in studies of adult cancers and medulloblastoma, however, previous in vitro findings have not been validated within the in vivo leptomeningeal niche [21, 22]. To enable reductionist studies of leptomeningeal biology in medulloblastoma, we developed and validated an in vitro model that recapitulates key features of the leptomeningeal niche. By co-culturing established and patient-derived medulloblastoma cell lines with primary human leptomeningeal fibroblasts, we demonstrate that meningeal cells promote tumor cell survival and proliferation under nutrient-deprived conditions. We further identify medulloblastoma models that reproducibly form laminar or nodular growth patterns in vitro and in vivo, revealing a cell-biological mechanism that explains this morphological divergence. This physiologically informed, experimentally validated model enables the dissection of the molecular determinants of leptomeningeal colonization and the identification of therapeutic vulnerabilities to prevent medulloblastoma dissemination.

## Materials and methods

### Cell culture

Cell lines utilized in this manuscript were routinely authenticated with short tandem repeat profiling and *Mycoplasma* testing (Laboratory Corporation of America). Cells used for experiments that were grown in suspension were routinely replaced within two months after being revived from liquid nitrogen storage. Primary cells used for experiments were used within 10 passages after being revived from liquid nitrogen storage. Medulloblastoma cell lines (and their molecular sub-group) used in this study include: D425 (Group 3), MED-411FH (Group 3), MED-2112FH (Group 3), and CHLA-01-MED (Group 4). D425 Med (D425) cells (RRID: CVCL_1275) were derived from a primary medulloblastoma tumor resection at Duke University Medical Center and obtained from the laboratory of Dr. Jeffery Leonard [23, 24]. Med-411FH (MED411) cells and Med-2112FH (MED2112) cells are cell lines derived from early passage patient-derived xenografts developed by the laboratory of Dr. Jim Olson and distributed by the Brain Tumor Resource Lab at Seattle Children’s Hospital [25]. CHLA-01-MED was obtained from American Type Culture Collection as described previously [26]. Human meningeal cells (HMC) (ScienCell Research Laboratories, Cat. #1400) are primary leptomeningeal cells derived from human donors. We have used > 5 distinct lots with similar results. D425 and HMC cells were cultured in DMEM (Corning, 10–013-CV) supplemented with 10% FBS (Atlanta Biologicals, S11150H). MED411, MED2112, and CHLA-01-MED cells were cultured in NeuroCult NS-A Basal Medium (Human) (Stemcell technologies, Cat. #05750) supplemented with NeuroCult NS-A Proliferation Kit (Human) (Stemcell technologies, Cat. #05751), human epidermal growth factor (20 ng/mL, ThermoFisher, AF-100–15-500UG) and human fibroblast growth factor-basic (20 ng/mL, ThermoFisher, 100-18B-50UG).

### HMC survival optimization

HMC were grown to confluence in 24-well plates. Media was exchanged for either basal growth media, serial dilutions of growth media with artificial CSF (ACSF, Tocris, Cat. No. 3525), ACSF + 60 mg/dL glucose, or DMEM/F12 (Gibco, Cat. #21,041,025). After 72 h, cell viability was assessed using alamarBlue (ThermoFisher, A50100), and cells were monitored overtime with long term live-cell imaging on an Incucyte SX5 (Sartorius).

### Medulloblastoma-leptomeningeal fibroblast co-culture

Human medulloblastoma cells and meningeal cells were co-cultured to generate an in vitro model that captures key interactions between tumor cells and the surrounding stroma of leptomeningeal metastasis within a reductionist setting. HMCs were seeded onto 0.4 μm transwell filters (Falcon, Product #353,090) or 1.5# glass coverslips (Electron Microscopy Sciences, 72,204–04) in 6- or 24-well dishes and cultured with DMEM supplemented with 10% FBS. After HMCs formed a confluent monolayer (48–72 h), the base media was removed and replaced with serum-free, growth-factor free DMEM/F12 containing medulloblastoma cells. For transwell experiments, DMEM/F12 was placed in the top and bottom wells.

### Cell-free extracellular matrix

HMC were grown to confluence on 1.5# coverslips for 72 h. Cell-free HMC-derived matrix was generated as previously described [27]. Medulloblastoma cells were plated onto coated coverslips for 48–72 h in their base media before processing for immunofluorescence.

### Animal studies

All animal procedures were approved by the Institutional Animal Care and Use Committee of the Abigail Wexner Research Institute at Nationwide Children’s Hospital (IACUC protocol AR15-00022). CB17-SCID mice [CB17SC scid − / − ; RRID: IMSR_TAC:cb17sc] were used for all in vivo experiments.

*Subcutaneous Tumor Model*: 6–8-week-old mice were anesthetized and injected subcutaneously in the flank with 5 × 10^5^ medulloblastoma cells, with or without 1 × 10⁶ HMCs. Tumor growth was monitored biweekly via caliper measurements. Mice were euthanized, and tumors were harvested upon a total tumor volume of 2000 mm^3^.

*Spontaneous Leptomeningeal Metastasis Model*: 6–8-week-old mice were anesthetized with isoflurane and placed in a stereotaxic apparatus (Kopf Instruments, Model 940). Intracerebellar injection of 8 × 10^4^ medulloblastoma cells in 3 µL of Neurocult media was performed at 2 mm lateral, 2 mm posterior, and 2.5 mm deep relative to lambda. Post-operative care included analgesia and daily monitoring. At neurological endpoint (rapid weight loss and an enhanced body condition score of less than 9, and/or neurological clinical signs including head tilts, seizures, and/or log rolling), mice were euthanized and processed for histology.

*Experimental Leptomeningeal Metastasis Model*: Medulloblastoma cells (2.5 × 10^4^ in 3 µL Neurocult media) were injected into the cisterna magna of 6–8-week-old mice under aseptic conditions. Post-operative care and endpoint monitoring mirrored the spontaneous model.

### Histology

Mice were euthanized at defined endpoints, sternotomy was performed, and mice were perfused with 20 ml of 0.9% saline injection (BD PosiFlush, cat #306,546) followed by 30 ml of neutral buffered formalin (NBF) (Sigma-Aldrich, HT5012-1CS) through the left ventricle. Heads and spinal columns were cleaned of connective tissue and placed in NBF at 4 °C for 48 h. Brains were dissected from skull and spines were decalcified in 10% EDTA (Elabscience, E-IR-R112) for 10 days at room temperature, and then processed for paraffin embedding (FFPE). For standard histological observation, FFPE sections were counterstained with hematoxylin and eosin via conventional methods.

### Immunohistochemistry and Immunofluorescence

Unstained 4 µm sections were deparaffinized with xylene and rehydrated through ethanol series. Heat-mediated antigen retrieval was performed with Tris–EDTA buffer (TE; pH 9) with 0.2% v/v Tween 20 (Fisher Bioreagents, BP337-100).

Cells growing on #1.5 glass coverslips or 0.4 µm filters were fixed in NBF for 10 min at room temperature. Following three rinses in 1 × Phosphate Buffered Saline (PBS) (Corning, 21–040-CM), coverslip or histological sections were then incubated for at least 1 h in blocking solution consisting of PBS, 0.2% triton100 (v/v) (Sigma, X-100-100 ml), and 3% bovine serum albumin (w/v) (Sigma, A-7888) at room temperature. Coverslips or histological sections were then incubated overnight in the following primary antibodies (diluted in blocking solution): hamster anti-Podoplanin (Developmental Studies Hybridoma Bank(DSHB), 8.1.1), rabbit anti-Fibronectin (Abcam, ab2413), rabbit anti-Collagen IV (Abcam, ab6586), rabbit anti-Synaptophysin (Abcam, ab32127), rabbit anti-GFAP (Abcam, ab68428), rabbit anti-CD31 (Abcam, ab182981), rabbit anti-pan laminin (Novus Biologicals, NB300-144), rabbit anti- Cleaved Caspase 3 (Cell Signaling Technologies-CST, 9664), rabbit anti- phospho-Histone Ser10 (CST, 53,348), goat anti-CD45 (R&D Systems, AF114), goat anti-OTX2 (R&D Systems, AF1979), sheep anti-S100A6 (R&D Systems, AF4584), or mouse anti-Vimentin (Abnova, SRL33). Following three rinses in PBS, sections or coverslips were incubated with appropriate AlexaFluor-labeled secondary antibodies (Invitrogen), DAPI (Invitrogen, D1306) and, where indicated, Phalloidin AlexaFluor 488, 568 or 647 (Invitrogen), or Wheat Germ Agglutinin (WGA) AlexaFluor 647 (Invitrogen, W11261) diluted in blocking solution for 1 h at room temperature. Following PBS rinses, coverslips and tissue sections were mounted in Fluoromount G (Invitrogen, 00495802).

### Microscopy and image analysis

Confocal microscopy images were obtained using a Crest-X-Light V3 spinning disk confocal on a Nikon Ti2-E inverted microscope, equipped with either a Hamamatsu ORCA Fusion camera or a Zeiss 800 laser scanning microscope, and featuring 4X, 20X, and 40X air objectives. Raw ND2 or czi files were imported into ImageJ for post-image processing and quantitation. All manipulation of images (brightness/contrast) were done uniformly to images. Mitotic (pH3 +) and apoptotic (CC3 +) were quantified by creating binary images with a manual threshold, then subjecting the binary images to the *Analyze Particles* function (size > 10µm^2^). Index was generated by dividing the cells (pH3 + or CC3 +) by the total number of medulloblastoma cells (OTX2 +). Disruption of the meningeal layer by medulloblastoma cells in co-culture was quantified using the *Percent Confluency* tool in the analysis software for the Incucyte SX5 live-cell imaging system following the co-cultures for a period of 7 days under serum deprived conditions. Spreading of medulloblastoma cells in co-culture was quantified by generating a binary image of OTX2 image and processed using the Skeletonize function. Skeletonized images were then quantified with *Analyze Skeleton* function. Percent spreading on HMC-derived ECM was quantified manually using the *Cell Counter* Plugin. H/E images were obtained with Aperio FL ScanScope digital slide scanner with 20X objective, and metastasis length was quantified using Aperio ImageScope Software (V12.4.3).

### Statistical analysis

All statistical analyses for in vitro assays and animal studies were performed using GraphPad Prism v10. The number of biological replicates (independent experiments or animals) is specified in the figure legends, along with the appropriate statistical tests and *P* values. Data are presented as the mean with error bars that represent the standard error of the mean.

## Results

### Pial fibroblast-medulloblastoma adhesion within the early metastatic niche

**Fig. 1:**
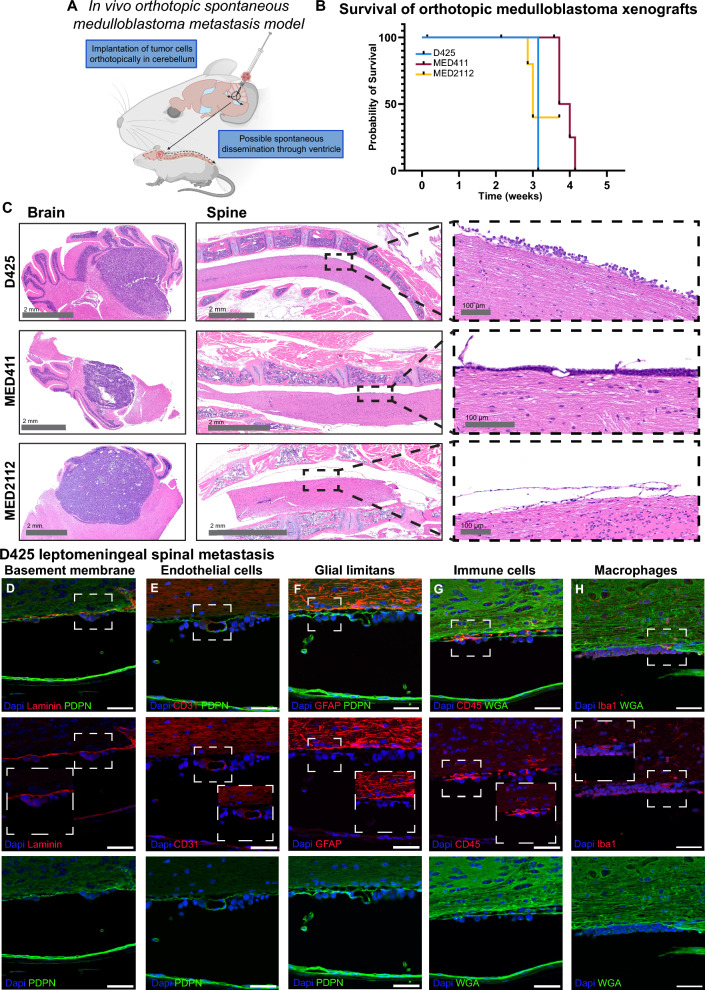
The early metastatic niche in medulloblastoma is composed of tumor cells and pial fibroblasts. **A** Schematic representation for generating spontaneous leptomeningeal dissemination. **B** Kaplan–Meier survival curve depicting the probability of survival across different orthotopic medulloblastoma models (n = 3/model). **C** Representative intracranial and spinal H/E for each medulloblastoma model. **D-H** Representative images of D425 tumor cells in relation to cells in leptomeningeal niches (Laminin = sub-pial basement membrane, CD31 = endothelial cells, PDPN = pial and arachnoid leptomeningeal fibroblasts, GFAP = astrocytes; glia limitans, CD45 = immune cells, Iba1 = myeloid cells/macrophages). WGA = Wheat germ agglutinin, marks stroma, including pial and arachnoid layers. n = 3. Scale bar = 50 µm

To guide the design of physiologically relevant in vitro models, we first generated models of the early leptomeningeal metastatic niche in vivo. Among medulloblastoma subgroups, Group 3 and Group 4 tumors most frequently disseminate to the leptomeninges[3]. Because Group 4 models are not widely available, we orthotopically injected three human Group 3 medulloblastoma cell lines (D425, MED411, and MED2112) into the cerebella of immunocompromised mice (6–8-week-old) to assess spontaneous metastasis (Fig. 1A). At humane endpoint, brains and spines were examined histologically for evidence of dissemination. Although all models formed robust primary tumors with comparable survival (Fig. 1B), small leptomeningeal lesions were detected only in D425 and MED411 (Fig. 1C). These findings highlight the potential limitations of orthotopic xenografts for studying leptomeningeal metastasis, as rapid primary tumor growth restricts the development of marked leptomeningeal disease.

We next defined the stromal architecture of the leptomeningeal niche in D425 spontaneous metastasis lesions. The cellular aspects of the leptomeninges include pial and arachnoid fibroblasts, penetrating blood vessels, a subpial basement membrane, tissue-resident and recruited immune cells, and the glial limitans [8, 9, 28–30]. Multiparameter immunohistochemistry identified pial and arachnoid fibroblasts (Podoplanin, PDPN), endothelial cells (CD31), basement membrane (laminin), glial limitans (GFAP), immune cells (CD45), and myeloid cells (IBA1). Tumor cells, identified by OTX2 expression (not shown for clarity), were consistently juxtaposed to pial fibroblasts (expressing PDPN), suggesting a preferential tumor–fibroblast interaction during early dissemination (Fig. 1D-H). Based on these observations and recently published data [31], we selected leptomeningeal fibroblasts as the primary stromal component for developing an in vitro co-culture model of the early leptomeningeal niche.

### Human leptomeningeal fibroblasts support medulloblastoma survival and growth

Leptomeningeal fibroblasts can be isolated from human donors and cultured in vitro [32]. To enhance the physiological relevance by mimicking the nutrient-deprived state of the CSF, we first defined the minimal media conditions capable of supporting primary human leptomeningeal fibroblasts (HMC). Standard HMC media (DMEM + 10% FBS) was diluted with increasing concentrations of artificial CSF (aCSF) containing physiologic glucose or replaced with serum- and growth factor–free DMEM/F12 (Supplemental Fig. S1A). While HMCs failed to survive in aCSF, confluent monolayers were maintained in DMEM/F12 (Supplemental Fig. S1B–C). Under these conditions, HMCs retained expression of pial markers including fibronectin, collagen IV, laminin, S100A6, and vimentin (Supplemental Fig. 1D). Thus, HMCs can be maintained under serum- and growth factor–free conditions that approximate the nutrient limitations of CSF.

**Fig. 2:**
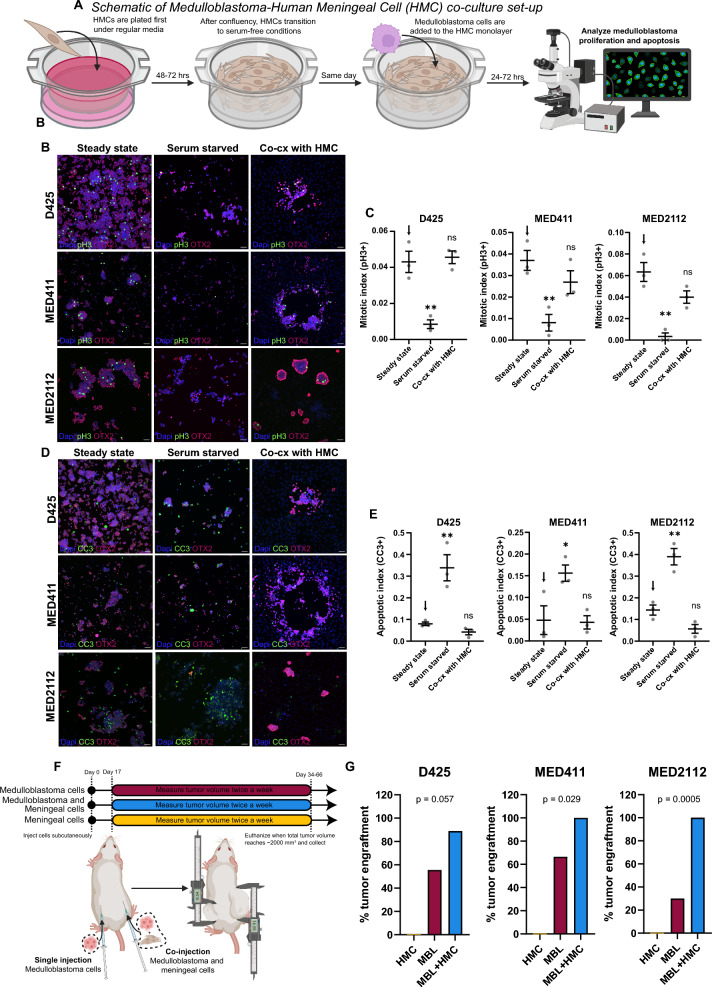
Meningeal cells promote medulloblastoma survival and growth. **A** Schematic of co-culture development. **B-C** Representative immunofluorescence images and quantitation of mitotic index (pH3 + cells/total tumor cells). One-way ANOVA with Dunnett’s multiple comparisons correction. n = 3 independent experiments, at least 300 cells/experiment. Scale bar = 50 µm. **D-E** Representative images of cleaved caspase 3 (CC3) and quantitation of apoptotic index (CC3 + cells/total tumor cells). One-way ANOVA with Dunnett’s multiple comparisons correction. *p < 0.05, **p < 0.01, ***p < 0.001, ****p < 0.0001. n = 3 independent experiments, at least 300 cells/experiment. Scale bar = 50 µm. **F** Schematic of subcutaneous implantation of medulloblastoma cells (+ human meningeal cells). **G** Bar graph depicting percentage of tumor engraftment for each subcutaneously implanted medulloblastoma model. Chi-square test, p-values are depicted in the bar graph for each model

Using these optimized conditions, we next tested whether HMCs promote medulloblastoma cell survival and proliferation in short-term co-culture (Fig. 2A). Under nutrient-deprived conditions, D425, MED411, and MED2112 cells exhibited reduced proliferation and increased apoptosis, as shown by phospho-H3 (S10) and cleaved caspase-3 (CC3) staining (Fig. 2B–E). Co-culture with HMCs restored proliferation and reduced apoptosis to near steady-state levels, indicating that leptomeningeal fibroblasts provide trophic support under nutrient-limited conditions.

To provide an orthogonal in vivo assessment of whether leptomeningeal fibroblasts directly enhance medulloblastoma growth under non-permissive conditions, we performed bilateral subcutaneous flank injections in immunocompromised mice, implanting tumor cells alone on one flank and tumor cells admixed with human meningeal cells (HMCs) on the contralateral flank of the same animal (Fig. 2F). This paired design was used to control inter-animal variability and was not intended to model the CNS microenvironment.

Across Group 3 models tested (D425, MED411, MED2112), co-injection with HMCs increased tumor establishment and growth kinetics compared to tumor-only injections (Fig. 2G**, **Supplemental Fig. S2 A-G). Importantly, injection of HMCs alone did not result in tumor formation, HMC were rare in end point tumors, and tumor cells demonstrated decreased apoptosis in co-injection tumors, indicating that the observed effects reflect support of tumor cell survival and expansion, rather than fibroblast-driven mass formation (Supplemental Fig. S2 H-J). Collectively, these findings complement the in vitro starvation assays by demonstrating that meningeal fibroblasts can promote medulloblastoma growth in vivo in an unfavorable, ectopic environment, independent of CNS-specific stromal or parenchymal cues.

### Distinct laminar and nodular morphologies reflect divergent modes of leptomeningeal colonization

**Fig. 3:**
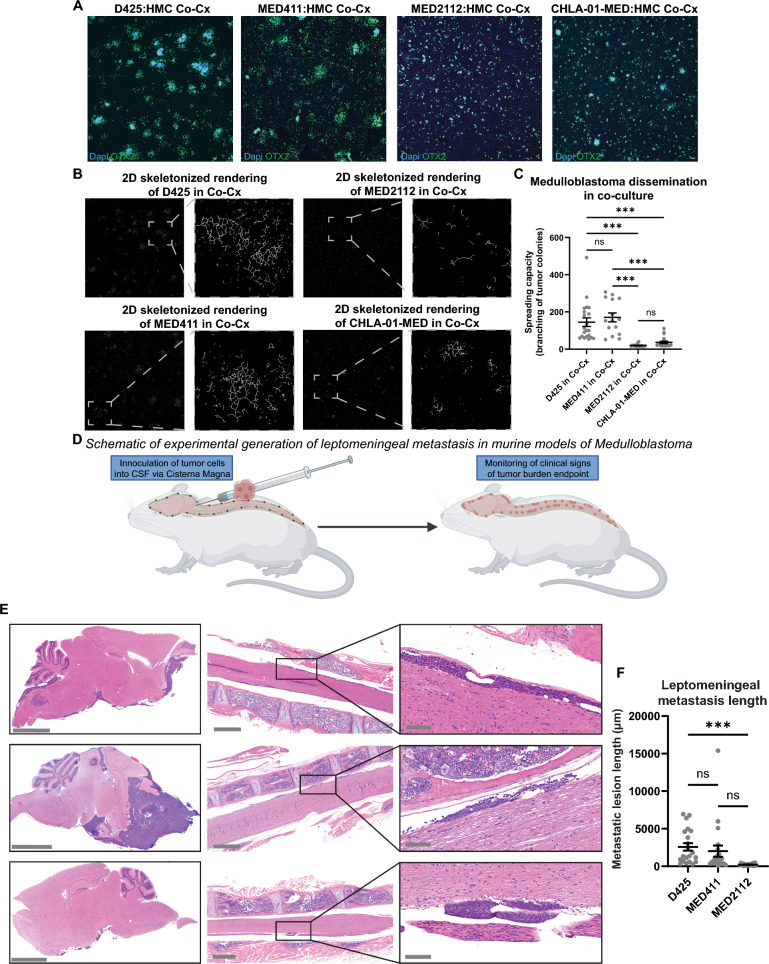
Distinct laminar and nodular growth patterns in vitro mirror leptomeningeal colonization phenotypes in vivo. **A** Representative images of established co-cultures for a panel of medulloblastoma cell lines. Scale bar = 50 µm. **B** Representative binary transformed images with skeletonization processing to quantify tumor cell spread. **C** Quantification of number of branch and junctions in co-culture per medulloblastoma model. n = 20 images per cell line from 3 independent experiments. Brown Forsythe and Welch’s ANOVA with Dunnett’s T3 multiple comparisons test. *p < 0.05, **p < 0.01, ***p < 0.001, ****p < 0.0001. **D** Schematic representation of generation of experimental leptomeningeal dissemination in murine models. **E** H&E of representative images of cranial and spinal leptomeningeal dissemination per medulloblastoma model from the spontaneous model of metastasis. **F** Quantification of spinal leptomeningeal lesion length per model. n = 3 mice/model. Brown Forsythe and Welch’s ANOVA with Dunnett’s T3 multiple comparisons test. *p < 0.05, **p < 0.01, ***p < 0.001, ****p < 0.0001

Medulloblastoma metastatic lesions adopt diffuse laminar or distinct nodular phenotypes in patients, though the mechanisms underlying these morphologies have not been elucidated due to lack of preclinical models. Having validated short-term co-culture feasibility, we next examined whether extended co-culture recapitulates the morphologic diversity of leptomeningeal metastases. A panel of medulloblastoma neurosphere lines—including Group 3 models (D425, MED411, MED2112) and one Group 4 model (CHLA-01-MED)—was co-cultured with HMCs under nutrient-deprived conditions for 7 days. Two reproducible and morphologically distinct growth patterns emerged: a **laminar phenotype** (D425, MED411) in which tumor cells disseminated from neurospheres and spread across the fibroblast monolayer, and a **nodular phenotype** (MED2112, CHLA-01-MED) characterized by compact, spherical aggregates with limited dispersion (Fig. 3A). To generate a quantitative metric of laminar versus nodular growth, OTX2 + tumor cell masks were converted to skeletonized representations, enabling measurement of branch number and extent as indicators of surface spreading (Fig. 3B-C). Laminar models (D425 and MED411) demonstrated significantly higher branching than nodular models (MED2112 and CHLA-01-MED), indicative of a more diffuse growth pattern in vitro.

We next sought to determine whether in vitro behaviors mirrored in vivo dissemination patterns. Because leptomeningeal disease progression is prevented by rapid primary tumor growth in spontaneous models (Fig. 1), we hypothesized that direct injection of tumor cells into CSF would allow for leptomeningeal tumor progression and evaluation of growth patterns between different models. Previous works have seeded medulloblastoma cells into CSF through injection of tumor cells into lateral ventricle or cisterna magna[31, 33, 34]. Medulloblastoma cells are thought to enter CSF through either direct invasion into 4th ventricle or following hematogenous dissemination[35]. Because the CSF within the 4th ventricle circulates directly into the cisterna magna, we chose intracisternal injections to inoculate D425, MED411, or MED2112 cells directly into the CSF. The resulting metastatic lesions recapitulated the in vitro patterns: laminar models (D425 and MED411) exhibited extensive surface coating of the intracranial and spinal meninges, whereas the nodular model MED2112 formed discrete, localized deposits (Fig. 3D). We quantified lesion length as a surrogate measure of laminar versus nodular growth and found that laminar lesions extended significantly farther along the pial surface than nodular lesions (Fig. 3E-F). We acknowledge the limitations of using quantitative metrics such as length to capture categorical morphologic features, mirroring the challenges faced clinically in assessing leptomeningeal disease [36, 37]. Collectively, the combination of in vitro and in vivo findings provide preclinical evidence for biologically distinct modes of leptomeningeal colonization.

### Laminar lesions disrupt the fibroblast layer and adhere to the subpial basement membrane

**Fig. 4:**
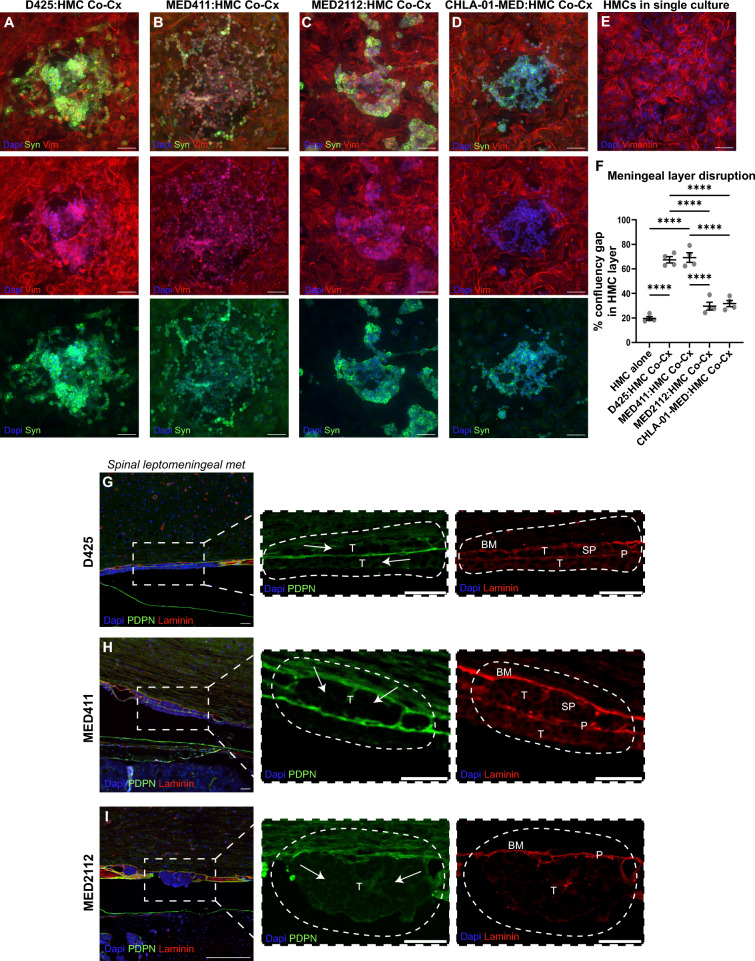
Laminar growing cells remodel the leptomeningeal pial layer during colonization. **A-D** Representative immunofluorescence images of medulloblastoma cells and HMC cells in co-culture for four different medulloblastoma models. **E** Representative immunofluorescence image of HMC monolayer in single culture. **F** Quantification of percentage of disruption of the HMC monolayer in single culture or co-culture with medulloblastoma cells after 7 days (n = 4 wells per model, 4 FOV per well averaged). One-way ANOVA with Tukey’s multiple comparisons testing. *p < 0.05, **p < 0.01, ***p < 0.001, ****p < 0.0001. **G-I** IHC of spinal leptomeningeal metastases for three medulloblastoma experimental metastases in three different models. Arrows = sub-pial gap created by laminar spreading cells (T = tumor, P = pial layer, SP = sub-pial gap, BM = basement membrane). Scale bar = 50 µm

We performed fixed, high resolution confocal microscopy and long-term live-cell imaging of HMC–tumor co-cultures to directly visualize tumor–fibroblast interactions in vitro. Both laminar and nodular medulloblastoma cells adhered rapidly to fibroblasts within hours of plating, however, laminar cells progressively displaced the fibroblast monolayer and spread along the underlying substrate, whereas nodular cells remained compact and adherent to pial cells. (Fig. 4A–F, Supplemental Fig. S3, Supplemental Videos S1, S2, S3 and S4.

In vivo immunohistochemistry of D425, MED411, and MED2112 leptomeningeal lesions corroborated these findings: laminar tumor cells penetrated the pial fibroblast layer to adhere directly to the laminin-positive subpial basement membrane. We did not observe invasion through the basement membrane or disruption of the glial limitans, consistent with clinical observations that parenchymal extension of disseminated disease is rare in medulloblastoma. In contrast, nodular lesions remained juxtaposed to intact pial fibroblasts (Fig. 4G–I). Thus, laminar and nodular lesions represent distinct modes of tumor–meningeal interaction, differing in their degree of fibroblast displacement and engagement with the subpial basement membrane.

### Adhesion to meningeal extracellular matrix distinguishes laminar from nodular cells

**Fig. 5:**
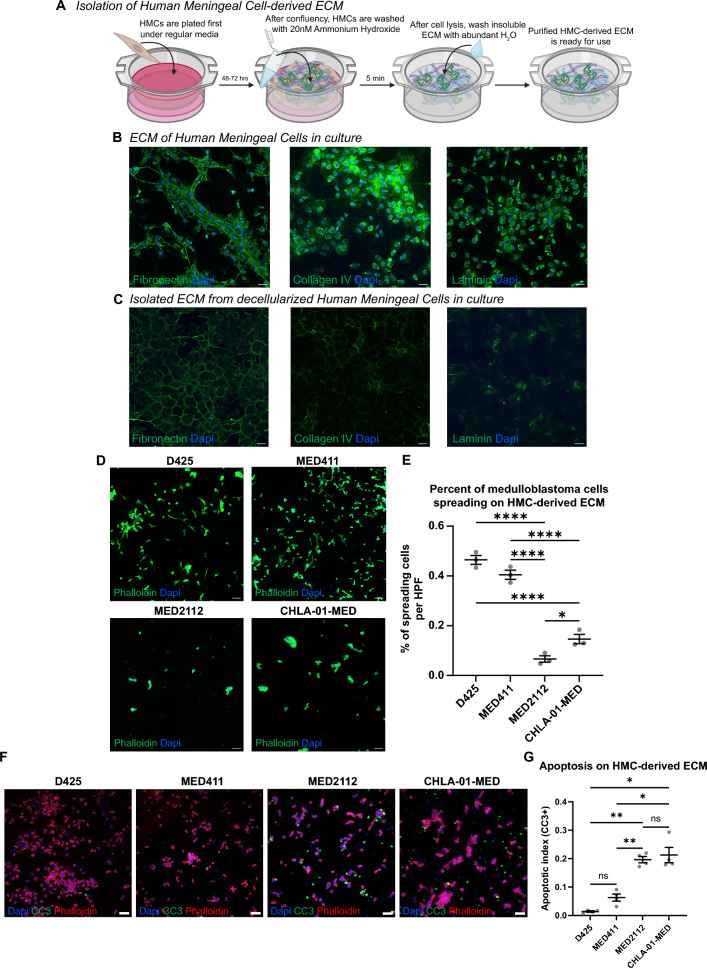
Medulloblastoma cells exhibit different patterns of spread on Human Meningeal Cell-derived extracellular matrix. **A** Schematic for generating cell-free HMC-derived extracellular matrix (ECM). **B-C** Representative images of non-extracted and extracted (cell-free) matrix. **B** Schematic depicting the process for isolating cell-free ECM from HMCs for biological assays. **C** Immunofluorescence images demonstrating that basement membrane proteins secreted by HMCs are maintained after cell lysis and ECM extraction (fibronectin, collagen IV and laminin). **D** Representative images of medulloblastoma cells cultured on cell-free HMC-extracted ECM. **E** Quantitation of % of cells spreading on HMC-derived ECM. n = 3 independent experiments, 300 cells/experiment. Ordinary one-way ANOVA with Tukey’s multiple comparison testing. *p < 0.05, **p < 0.01, ***p < 0.001, ****p < 0.0001. **F** Representative images of CC3 staining on cell-free HMC-extracted ECM. **G** Quantitation of apoptotic index. n = 4 independent experiments, 300 cells/experiment. Scale bar = 50 µm. Brown Forsythe and Welch’s ANOVA with Dunnett’s T3 multiple comparisons test. *p < 0.05, **p < 0.01, ***p < 0.001, ****p < 0.0001

We next sought to determine how medulloblastoma cells interact with extracellular matrix (ECM) derived from leptomeningeal fibroblasts given the differential patterns of spread observed in previous experiments. To directly quantify medulloblastoma behavior on pial ECM, we generated cell-free HMC ECM by culturing HMC on coverslips and performing a decellularization method described previously[27] (Fig. 5A–C). Decellularized ECM retains both the structure and biological properties of leptomeningeal-deposited ECM, even in the absence of leptomeningeal fibroblasts (noted by the absence of nuclear DAPI staining). D425, MED411, MED2112, and CHLA-01-MED were plated on decellularized HMC-derived ECM for 24 h. Laminar cells (D425 and MED411) adopted an elongated morphology with filopodia-like projections characteristic of spreading and migration. In contrast, nodular cells (MED2112 and CHLA-01-MED) retained cortical actin organization and failed to spread on HMC-derived ECM (Fig. 5D-E). Lastly, nodular cells exhibit significantly increased apoptosis when cultured on HMC-derived extracellular matrix, indicating reduced fitness and impaired adaptation to ECM-dependent growth conditions (Fig. 5F-G).

**Fig. 6:**
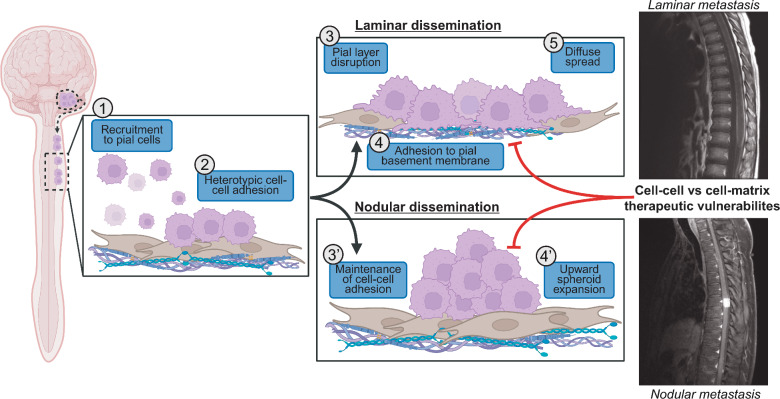
Schematic depicting the proposed model for medulloblastoma leptomeningeal colonization: Medulloblastoma cells are recruited **(1)** and directly adhere to leptomeningeal pial cells **(2)**. Laminar disseminating cells disrupt the pial fibroblast layer **(3)** to expose and adhere to the underlying basement membrane **(4)**. Cell–matrix adhesion enables the spread of cells along the brain and spinal cord to form laminar, diffuse metastases. Nodular lesions remain adherent through cell–cell interactions between medulloblastoma cells and pial fibroblasts **(3’)** and expand outward without diffuse spread along the leptomeninges **(4’)**

Together, these observations suggest that laminar and nodular phenotypes reflect fundamentally distinct adhesion behaviors on meningeal-derived ECM. Laminar cells actively engage and remodel the pial surface, whereas nodular cells remain cohesive and non-spreading. These cellular behaviors define two complementary strategies of leptomeningeal colonization that parallel the morphologic heterogeneity observed in patients (Fig. 6).

## Discussion

Leptomeningeal metastasis remains the principal cause of treatment failure and death in children with medulloblastoma, yet its underlying biology has remained elusive. Here, we developed an in vitro model that faithfully mirrors the morphologic diversity of leptomeningeal metastasis observed in vivo. Using this system, we identify two distinct modes of leptomeningeal colonization—laminar and nodular—that arise from differential tumor–stroma adhesion mechanisms. Our findings build upon previous iterations of in vitro meningeal and ECM medulloblastoma models by providing in vivo validation[22, 38, 39]. Future studies will define the molecular mechanisms that distinguish laminar and nodular phenotypes and determine whether cellular plasticity exists between these states. Integrating in vitro co-culture systems with both spontaneous and experimental leptomeningeal metastasis models establishes a versatile platform for delineating the stage-specific roles of metastatic mediators; whether they govern invasion and dissemination from the primary site, colonization of the leptomeninges, or subsequent progression within the leptomeningeal niche [34, 40–42]. Additionally, understanding the pathways that regulate these phenotypic transitions may have translational significance, given differential therapeutic sensitivities between clinical phenotypes [12, 43]. The model described here provides a tractable platform to investigate therapeutic response as a function of leptomeningeal colonization architecture.

### The leptomeningeal niche as a trophic microenvironment

A recent landmark paper from the Taylor group identified medulloblastoma-induced changes to the leptomeningeal environment during colonization and established the importance of tumor-host interactions in leptomeningeal metastasis [31]. Our findings agree with those observations by providing direct evidence that human meningeal cells provide trophic support to medulloblastoma cells. Co-culture of medulloblastoma cells with HMC rescued tumor cell proliferation and suppressed apoptosis under nutrient-deprived conditions, demonstrating that fibroblast-derived cues compensate for the metabolic limitations of CSF. Similar trophic effects have been described in lung and bone marrow metastases, suggesting that stromal support is a conserved feature of the metastatic niche across organ systems [44]. By defining leptomeningeal fibroblasts as key mediators of this process, our study establishes a cellular foundation for dissecting paracrine and adhesion-dependent interactions that sustain leptomeningeal metastasis.

### Modeling implications and future directions

Traditional xenograft models are limited in their ability to capture the temporal or spatial features of leptomeningeal dissemination due to rapid intracerebellar tumor growth and limited sampling of metastatic lesions. The co-culture system described here complements these in vivo models by offering a reductionist platform for mechanistic dissection and therapeutic testing. Integration of single-cell and spatial transcriptomic profiling will enable mapping of tumor–stroma signaling networks and identification of context-specific dependencies. In parallel, the model provides an experimental framework for evaluating compounds that disrupt adhesion-dependent survival. Future studies will delineate the mechanisms that distinguish cell–cell from cell–matrix adhesion in nodular and laminar metastases, as disrupting these interactions may represent context-specific therapeutic vulnerabilities within the leptomeningeal niche.

While our model captures key aspects of the leptomeningeal niche, it lacks other microenvironmental components, such as immune or vascular cells, that may modulate colonization dynamics and progression [31, 35, 45, 46]. Incorporating these elements in future iterations will further enhance physiologic relevance. The models used in this study were predominantly Group 3 medulloblastoma lines with MYC amplification, reflecting both their aggressive and metastatic phenotype and their availability [47]. Future work will extend these studies to WNT-, SHH-activated, and non-MYC–amplified Group 3/4 tumors, ideally using patient-derived xenografts or primary human specimens, to capture the full molecular spectrum of medulloblastoma leptomeningeal metastasis. Ultimately, coupling these advanced models with clinical and genomic data from patient-derived metastasis samples will enable a more complete understanding of metastatic evolution in medulloblastoma.

## Conclusions

Our study delineates modes by which medulloblastoma colonizes the leptomeninges. We identify differential interactions with fibroblast-derived extracellular matrix as a key determinant in influencing metastatic phenotype. This framework establishes the leptomeningeal niche as both a driver of metastatic persistence and a therapeutic vulnerability, offering new avenues for targeting the most lethal stage of pediatric medulloblastoma.

## Supplementary Information

Additional file1 (MP4 17132 KB)

Additional file2 (MP4 23175 KB)

Additional file3 (MP4 19667 KB)

Additional file4 (MP4 20418 KB)

Additional file5 (TIF 14664 KB)

Additional file6 (TIF 14477 KB)

Additional file7 (TIF 3454 KB)
