# Supplementary figures and images for: Meningeal-tumor interactions define distinct modes of leptomeningeal colonization in Group 3 medulloblastoma
Source: Acta Neuropathol Commun. 2026 Apr 1;14:108. doi: 10.1186/s40478-026-02253-7 (PMC13169881; doi:10.1186/s40478-026-02253-7)

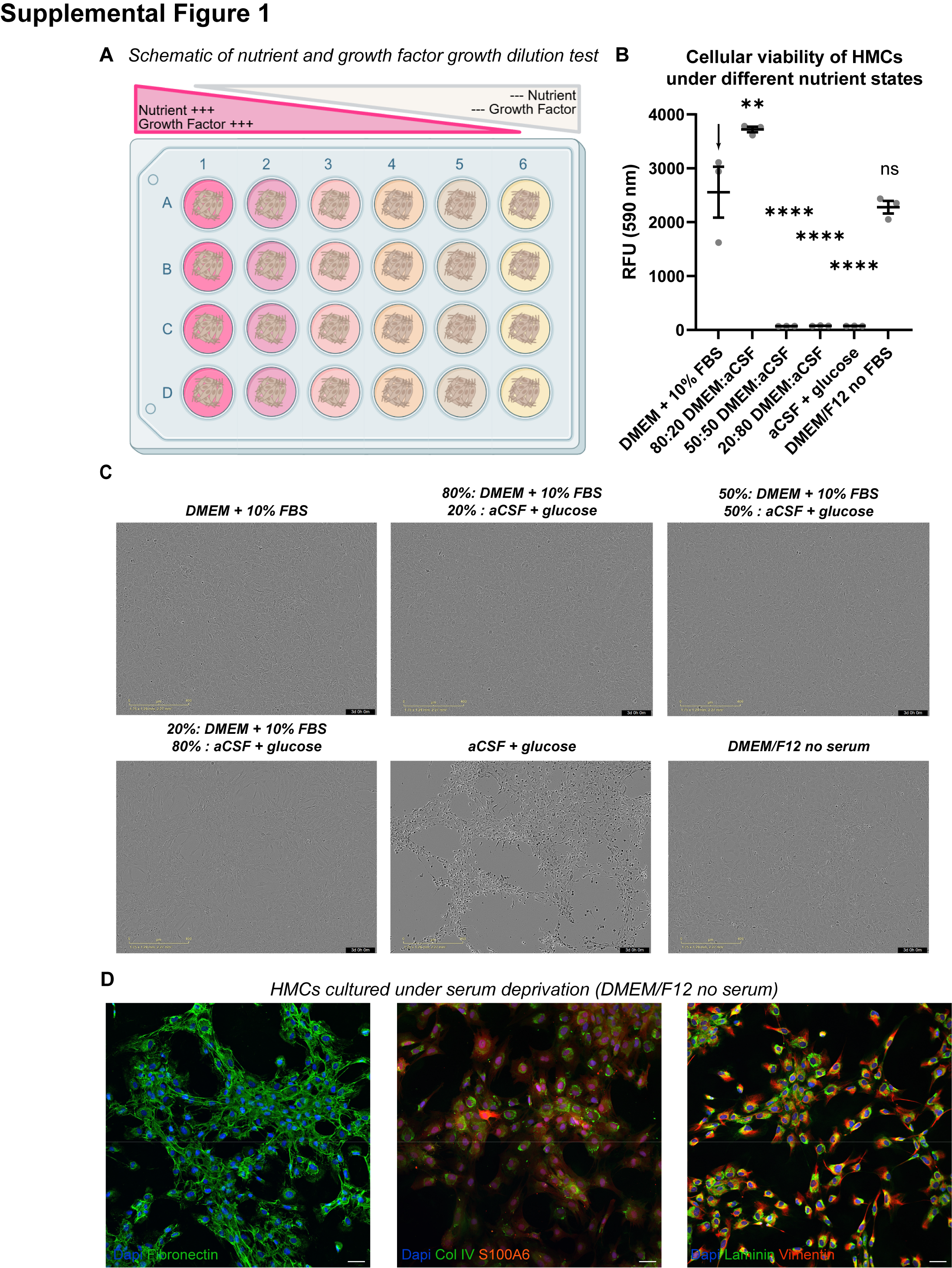

Supplement: Supplementary file 5 — Additional file5 (TIF 14664 KB) [file 40478_2026_2253_MOESM5_ESM.tif]

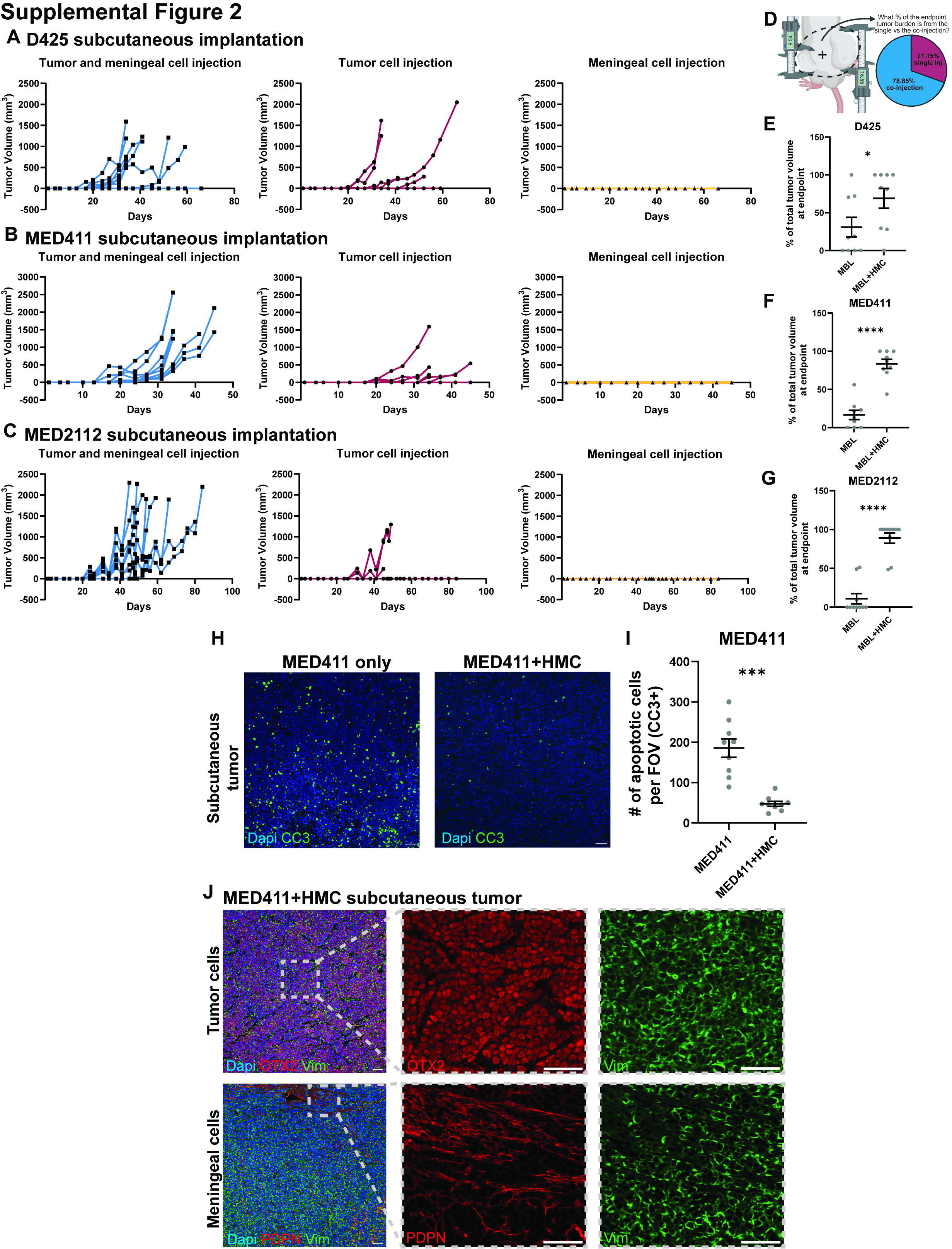

Supplement: Supplementary file 6 — Additional file6 (TIF 14477 KB) [file 40478_2026_2253_MOESM6_ESM.tif]

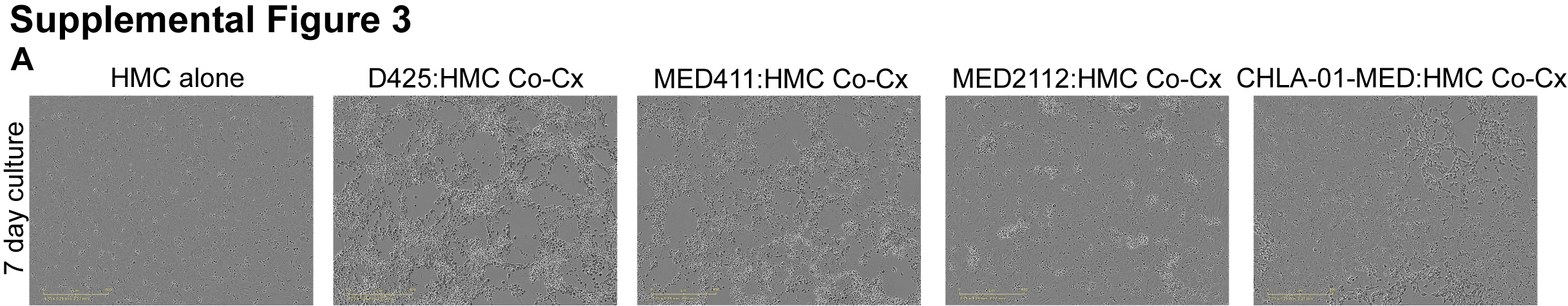

Supplement: Supplementary file 7 — Additional file7 (TIF 3454 KB) [file 40478_2026_2253_MOESM7_ESM.tif]
